# Establishment of a normal-derived estrogen receptor-positive cell line comparable to the prevailing human breast cancer subtype

**DOI:** 10.18632/oncotarget.14554

**Published:** 2017-01-06

**Authors:** Branden M. Hopkinson, Marie C. Klitgaard, Ole William Petersen, René Villadsen, Lone Rønnov-Jessen, Jiyoung Kim

**Affiliations:** ^1^ Department of Cellular and Molecular Medicine, University of Copenhagen, DK-2200 Copenhagen, Denmark; ^2^ Danish Stem Cell Centre, University of Copenhagen, DK-2200 Copenhagen, Denmark; ^3^ Department of Biology, University of Copenhagen, DK-2100 Copenhagen, Denmark

**Keywords:** human breast, breast cancer, estrogen receptor, immortalized luminal cells

## Abstract

Understanding human cancer increasingly relies on insight gained from subtype specific comparisons between malignant and non-malignant cells. The most frequent subtype in breast cancer is the luminal. By far the most frequently used model for luminal breast cancer is the iconic estrogen receptor-positive (ER^pos^) MCF7 cell line. However, luminal specific comparisons have suffered from the lack of a relevant non-malignant counterpart. Our previous work has shown that transforming growth factor-β receptor (TGFβR) inhibition suffices to propagate prospectively isolated ER^pos^ human breast luminal cells from reduction mammoplasties (HBEC). Here we demonstrate that transduction of these cells with hTERT/shp16 renders them immortal while remaining true to the luminal lineage including expression of functional ER (iHBEC^ERpos^). Under identical culture conditions a major difference between MCF7 and normal-derived cells is the dependence of the latter on TGFβR inhibition for ER expression. In a breast fibroblast co-culture model we further show that whereas MCF7 proliferate concurrently with ER expression, iHBEC^ERpos^ form correctly polarized acini, and segregate into proliferating and ER expressing cells. We propose that iHBEC^ERpos^ may serve to shed light on hitherto unappreciated differences in ER regulation and function between normal breast and breast cancer.

## INTRODUCTION

Although human breast cancer was one of the first cancer forms to receive precision medicine based on molecular ER profiling, a number of questions pertinent to the insurgence and treatment failure of this disease remain largely unanswered. One of the most puzzling discoveries is the apparent master switch between quiescence in normal ER^pos^ breast epithelial cells and proliferation in ER^pos^ breast cancer cells with concurrent increasing failure to down-regulate ER [1, 2]. Likewise, the opposite scenario, i.e. the evolution of a complete receptor-negative breast cancer leading to *de novo* resistance to anti-estrogen treatment also poses a considerable challenge and remains poorly understood [3].

Our current understanding of the regulation of ER expression and the mechanism of action of estrogen in human breast cancer almost exclusively relies on experiments with one cell line, MCF7, established from a metastatic lesion more than four decades ago [4]. Accordingly, MCF7 has received more than twenty five thousand hits in PubMed (for review see [5]). Other than being a widely used model for ER^pos^ breast cancer MCF7 also represents luminal B breast cancer which aside from being very proliferative is characterized by exhibiting a phenotype reminiscent of the luminal lineage in the normal human breast [6, 7]. This lineage is characterized by an almost universal expression of the simple cytokeratins K7, K8, K18, and K19 and the concomitant overall absence of basal cytokeratins K5, K6, K14, and K17 (for review see [8]). It is also characterized by expression of a highly glycosylated sialomucin encoded by MUC1 [9, 10].

Non-malignant equivalents to breast cancer subtypes in general have proven to be extremely valuable in understanding breast cancer evolution and in the search for precision drug targets [11–15]. However, when it comes to the by far most frequent luminal cancer, the ER^pos^, a non-malignant equivalent does not exist [7, 16]. Spontaneously immortalized cell lines such as HMT3522 [17] and MCF10A [18] are better counterparts for basal-like breast cancer and they are negative for keratin K19 and ER [19], and telomerase immortalized human breast epithelial cells continue to express basal keratin K14 and p63 [13]. Likewise, while modifying p53 and Rb by SV40 or E6/E7 transfection leads to established cell lines with luminal characteristics, functional ER expression at the protein level has not been accomplished [20, 21].

Recently, we isolated and cultured human breast ER^pos^ cells which remained responsive to estrogen and showed that inhibition of TGFβR signaling was key to release of ER^pos^ cells from growth restraint [22]. Extended culture was obtained by transduction with hTERT/shp16. Here we describe iHBEC^ERpos^ which are remarkably similar in phenotype to MCF7 by critical lineage markers and ER expression. We compare the functional properties of iHBEC^ERpos^ and MCF7 under identical culture conditions which offers a unique opportunity to dissect at the molecular level the aberrations associated with malignant transformation of the most frequent breast cancer subtype.

## RESULTS

### A luminal ER^pos^ cell line, iHBEC^ERpos^, is established from hTERT/shp16 transduction of normal breast ER^pos^ cells

**Figure 1:**
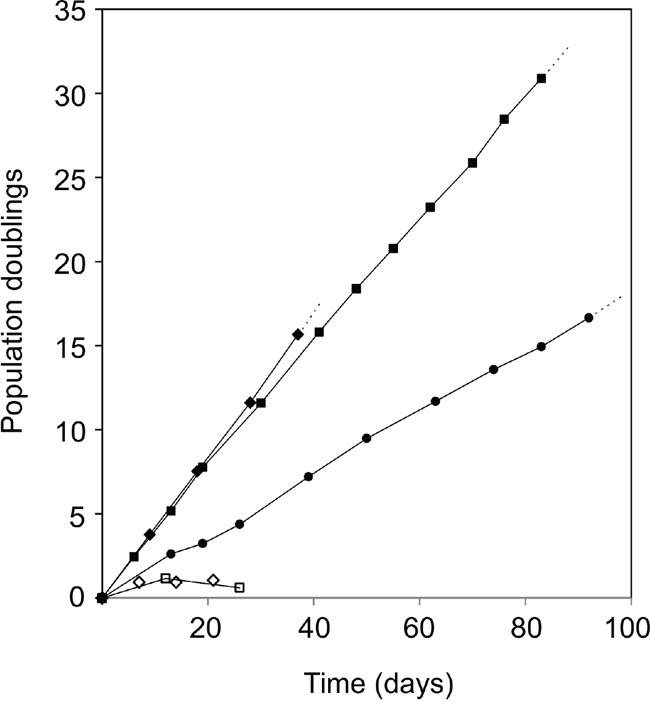
Infinite lifespan of hTERT/shp16 transduced iHBEC^ERpos^ cells Population doublings as a function of time in culture of iHBEC^ERpos^ cells (passage 28-36, circles) and MCF7 cells (passage 309-319, squares) upon switch to TGFβR2i-1. Both cell lines have infinite lifespans under these conditions. MCF7 cannot grow in TGFβR2i (open diamonds, individual time points), but readily adapt to TGFβR2i-1 (closed diamonds) and stop growing and cannot be passed more than twice when switched back to TGFβR2i (open squares).

**Figure 2:**
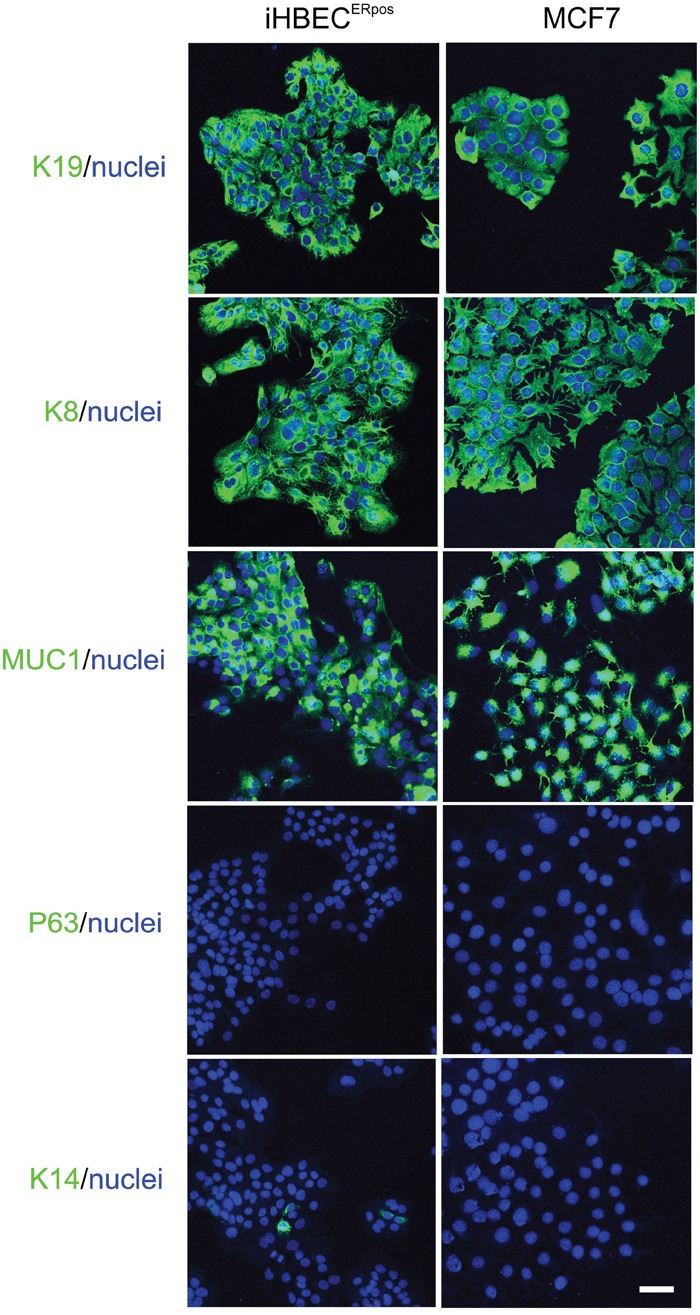
Characterization of hTERT/shp16 transduced iHBEC^ERpos^ cells Immunofluorescence staining of iHBEC^ERpos^ and MCF7 cells with key breast lineage markers luminal keratin K19, K8 and MUC1 and myoepithelial p63 and K14 (green). Nuclei are counterstained with DAPI (blue). Note the striking similarity with respect to epitheloid morphology and luminal profile. Bar: 50μm.

**Figure 3:**
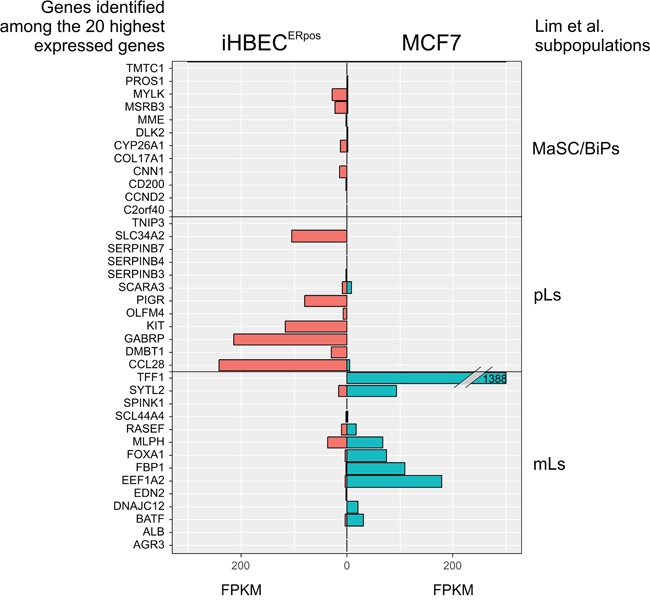
iHBEC^ERpos^ cells exhibit an expression profile reminiscent of luminal progenitors Expression profiles of twenty highest ranking markers in each group of mammary stem cells/bipotent cells (MaSC/BiPs), luminal progenitors (pLs), and mature luminal cells (mLs), respectively, according to the Lim classification [23] as compared to gene expression profiles of iHBEC^ERpos^ and MCF7 by RNA-Seq. Twelve, twelve and fourteen genes in each group, respectively, were informative. Whereas mature luminal genes are highly expressed in MCF7, luminal progenitor genes are pronounced in iHBEC^ERpos^ cells. X-axis indicates FPKM values, as calculated by gene expression levels (Fragments Per Kilobase Of Exon Per Million Fragments Mapped; FPKM in iHBEC^ERpos^, red, and MCF7, blue), and Y-axis indicates each subpopulation of genes present in our RNA-Seq dataset.

Using a high titer sequential retroviral transduction protocol we transduced reduction mammoplasty-derived, prospectively sorted CD166^high^/CD117^low^ luminal cells with a combination of hTERT and shp16 [22] and monitored proliferation of these cells over a few months. iHBEC^ERpos^ was established which, unlike the non-transduced control, could be expanded continuously without undergoing crisis. Since our aim was to establish a common ground for comparison between iHBEC^ERpos^ and MCF7, we tested the ability of MCF7 to grow under similar conditions. TGFβR2i did, however, not support growth of MCF7 (Figure 1). Therefore, we tested which growth factors in TGFβR2i should be omitted to allow growth, and identified cholera toxin and hydrocortisone as inhibiting factors. As we had previously observed that substitution of epidermal growth factor with amphiregulin supported ER expression and function in normal cells, this modification was included in the modified medium, referred to as TGFβR2i-1. MCF7 was easily adapted to grow under these conditions (Figure 1). Switching back to TGFβR2i again inhibited growth of MCF7, underscoring that MCF7 indeed cannot grow in TGFβR2i (Figure 1). iHBEC^ERpos^ cells easily adapted to TGFβR2i-1, and thus, MCF7 cells and iHBEC^ERpos^ cells both grew well on TGFβR2i-1 (Figure 1). This opened for direct comparisons under identical conditions. Normal luminal epithelium and luminal breast cancer have been successfully characterized by expression of keratin K8, K19 and MUC1 and lack of expression of keratin K14 and p63. We found that iHBEC^ERpos^ essentially aligned with these criteria and thus critically resembled MCF7 cells by lineage (Figure 2). A more accurate position of iHBEC^ERpos^ in the breast hierarchy was obtained by use of Lim et al.'s gene expression data [23]. Comparison of the RNA-Seq expression profile of iHBEC^ERpos^ with the available top twenty highest expressed genes for each of the epithelial subpopulations in Lim et al.'s differentiation hierarchy - ranging from mammary stem cells/bipotent progenitors (MaSC/BiPs) through luminal progenitors (pLs) to mature luminal cells (mLs) - revealed that iHBEC^ERpos^ cells most likely are equivalent to luminal progenitors (Figure 3).

### iHBEC^ERpos^ are able to recapitulate normal-like behavior in three-dimensional rBM

**Figure 4:**
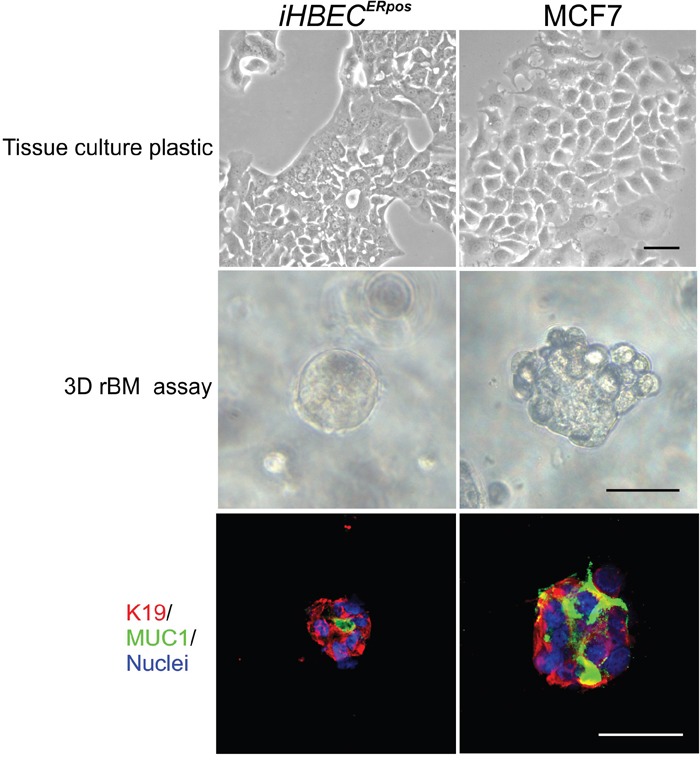
iHBEC^ERpos^ cells are normal-like by the rBM assay Phase contrast micrographs of iHBEC^ERpos^ (left column) and MCF7 cells (right column) on tissue culture plastic (upper panel) and in 3D rBM gels at day 8 (middle panel). Whereas both iHBEC^ERpos^ and MCF7 cells in monolayer culture are typically epitheloid, inside rBM gels iHBEC^ERpos^ are capable of forming acinus-like spheres with a central lumen while MCF7 cells grow as solid irregular colonies. Cryostat sections of rBM gels (lower panel) stained with MUC1 (green), K19 (red) and nucleus counterstain (blue) show that iHBEC^ERpos^ are correctly polarized while MCF7 fail to polarize. Bar: 50μm.

To establish functional evidence for the non-malignant nature of iHBEC^ERpos^, we employed our original reconstituted basement membrane (rBM) assay [24]. Whereas both iHBEC^ERpos^ and MCF7 cells formed similar epitheloid sheets in monolayer culture, in three-dimensional rBM, many iHBEC^ERpos^ cells remained as single cells and eventually died, but some were capable of forming clonal acinus-like spheres (33.2% +/- 0.9) with a central lumen (Figure 4). By comparison MCF7 cells grew into larger clusters of cells (Figure 4). Staining of the sectioned rBM gels showed that acinus-like iHBEC^ERpos^ cells were luminally restricted and correctly polarized with apical expression of MUC1 (Figure 4), while MCF7 cells remained unpolarized (Figure 4). ER expression was lost in both lines by exposure to rBM. Upon this initial characterization of the two cell lines, we focused our subsequent analyses on comparing iHBEC^ERpos^ with MCF7 cells with respect to their response to the principal female sex hormone, estradiol.

### ER expression in iHBEC^ERpos^ is subject to TGFβR regulation

**Figure 5:**
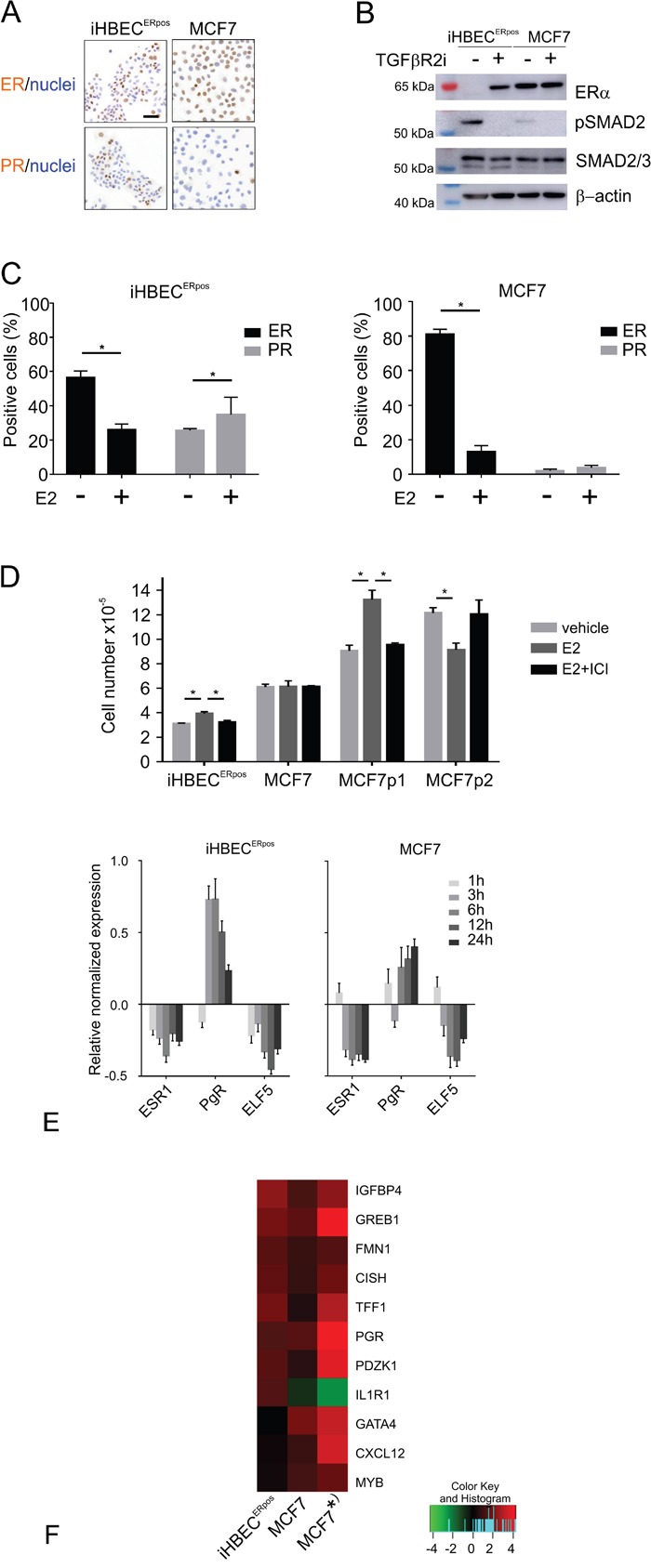
iHBEC^ERpos^ and MCF7 cells respond differently to TGFβR inhibitors and estrogen A. Immunoperoxidase staining of iHBEC^ERpos^ (left column) and MCF7 (right column) stained with ER (upper panel) and PR (lower panel) and counterstained with hematoxylin. B. Western blotting of proteins extracted from iHBEC^ERpos^ cells or MCF7 at day 6 upon omission of TGFβR inhibitors (-) or in continuous TGFβR2i-1 (+), incubated with antibodies recognizing ERα (upper panel), phosphorylated SMAD2 (pSMAD2, second panel), SMAD2/3 (third panel) and loading control β-actin (lower panel). While iHBEC^ERpos^ cells upregulate pSMAD2 and lose ER expression upon omission of TGFβR inhibitors, MCF7 upregulate pSMAD2 without concurrent regulation of ER. C. Quantification of ER (black bars) and PR (grey bars) expression by immunoperoxidase staining of iHBEC^ERpos^ (left panel) and MCF7 cells (right panel) cultured with vehicle or estrogen (E2) shows that ER is downregulated in both lines upon exposure to estrogen, and PR is significantly upregulated in iHBEC^ERpos^ (asterisks indicate significance by Student's T-test, p<0.05). D. Cell number after 7 days in quaduplicate cultures of iHBEC^ERpos^ and MCF7 plated at 6,000 and 4,000 cells/cm2, respectively, and exposed to vehicle (light grey bars), to estrogen (10-8M, dark grey bars) without or with estrogen receptor antagonist (10-8M and 10-9M ICI 182,780, respectively, black bars). Two different lines of the MCF7 parental line plated at 4,000 cells/cm2 in triplicate grown in standard medium exposed to estrogen (10-8M) without or with ICI 182,780 (10-7M) demonstrate growth stimulation in the immediate origin of the TGFβR2i-1-adapted subline (MCF7p1) and inhibition in response to estrogen in a line grown in another laboratory (MCF7p2). Bars indicate mean and standard deviation and technical variation. Asterisks indicate significance (p< 0.05; Student's T-test, two-tailed). E. RT-qPCR of ESR1, PgR and ELF5 gene expression levels in iHBEC^ERpos^ (left panel) and MCF7 (right panel) upon exposure to estrogen for 1h, 3h, 6h, 12h, and 24h, respectively. Y-axis indicates relative normalized gene expression levels compared to vehicle-treated samples in log2-scale. F. Heat map of fold difference assessed by RNA-Seq in expression of genes regulated in iHBEC^ERpos^ (left), MCF7 (middle) and a previously published dataset on MCF7 grown in standard medium (* right; [28]). Similarity in estrogen-regulated gene expression profile between iHBEC^ERpos^ and MCF7 includes IGFBP4, GREB1, FMN1, CISH, TFF1, PGR, PDZK1, and differences include the genes GATA4, CXCL12 and MYB, which are upregulated in MCF7 only, and IL1R1, which is upregulated in iHBEC^ERpos^, but downregulated in MCF7. Color key indicates fold difference in log2 scale.

To assess the fundamental precondition for estrogen action we first stained for canonical ERα. We found distinct nuclear ER staining in about half of the iHBEC^ERpos^ cells and in the majority of MCF7 cells (Figure 5A and 5C). To ensure that the observed iHBEC^ERpos^ phenotype reflects that of non-immortalized cells under similar conditions, we further verified that EpCAM^high^/CD271^low^/CD166^high^/CD117^low^ cells in early culture could be expanded and retained ER expression in TGFβR2i-1. We have previously shown that ER expression in normal breast epithelial cells is dependent on continuous TGFβR inhibition [22], and we therefore addressed whether TGFβR inhibitors affected SMAD signaling and ER expression in MCF7 in a similar way. Within six days of omission of TGFβR inhibitors, iHBEC^ERpos^ upregulated pSMAD2 and downregulated ER completely (from 44 +/- 5% ER-positive cells with inhibitors to 0% upon omission; Figure 5B), while ER expression in MCF7 cells was unaffected (87 +/- 4 % ER-positive cells with inhibitors and 90 +/- 7 % without inhibitors, n=3 × 100 cells, not significant by Student's T-test). Apparently, however, the lack of ER regulation in MCF7 was not due to insensitivity to TGFβR inhibition, since pSMAD2, albeit to a relatively modest level, was induced upon omission of TGFβR inhibitors (Figure 5B). The results suggest that in MCF7 ER expression is independent of TGFβR signaling.

### Estrogen-regulated genes differ between iHBEC^ERpos^ and MCF7

The presence of elements of a functional ER signaling pathway in iHBEC^ERpos^ and MCF7 cell lines was further demonstrated by staining for ER and progesterone receptor (PR) in response to estrogen (Figure 5C). While both lines down-regulated ER expression upon stimulation with estrogen, iHBEC^ERpos^ significantly upregulated PR protein expression (Figure 5C). We next assessed the growth response to estrogen with or without the estrogen receptor antagonist, ICI-182,780 (Figure 5D). While estrogen-induced proliferation was completely abrogated by ICI-182,780 in iHBEC^ERpos^, MCF7 did not exhibit a proliferative response in TGFβR2i-1 (Figure 5D). That MCF7 sublines may exhibit different growth responses to estrogen is not unprecedented. Here, MCF7 immediately prior to adaptation to TGFβR2i-1 readily responded, whereas another line of the parental MCF7 line grown in another laboratory exhibited growth inhibition (Figure 5D). At the molecular level we found elements of an estrogen response common between iHBEC^ERpos^ and MCF7, but more importantly also subtle differences. Based on an RT-qPCR time course of key estrogen-regulated genes, we found that a six hour-exposure to estrogen was the optimal time point for further RNA-Seq expression analysis of the two cell lines (Figure 5E). To identify differentially expressed genes regulated by estrogen, a robust bioinformatics method, NOISeq [25], was performed. Among statistically significant most-up-regulated genes in iHBEC^ERpos^ (fold difference >2 and probability >0.7), we found well known estrogen-regulated genes, such as IGFBP4 and GREB1 (Figure 5F) [26, 27]. These genes have physiological roles in steroid hormone responsive tissues, and were also upregulated in the present MCF7 cells as well as in an alternative dataset on the MCF7 estrogen response (Figure 5F) [28]. Three genes, GATA4, CXCL12 and MYB, were significantly upregulated in MCF7, while changes in expression were not observed in iHBEC^ERpos^. These are all estrogen regulated genes that have been implicated in breast cancer evolution [29–31]. Moreover, a cytokine binding receptor, IL1R1, found by others [28] to be significantly down-regulated in MCF7 was also downregulated in MCF7 here (1.5 fold), but was significantly upregulated in iHBEC^ERpos^. These findings implicate that availability of normal breast ER^pos^ cells may reveal important endocrinological differences between normal and cancer.

### A relevant stromal microenvironment segregates proliferating and ER-expressing normal cells

**Figure 6:**
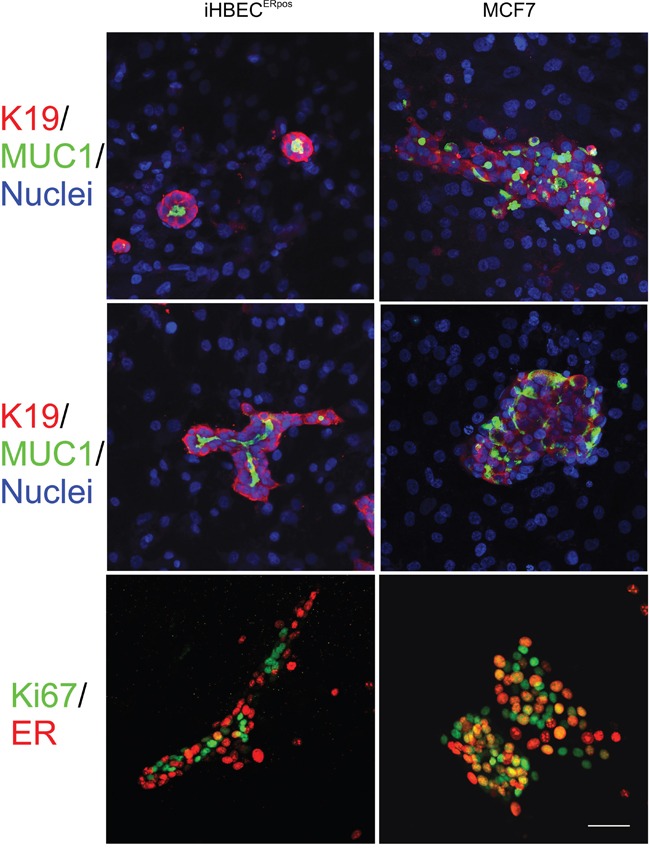
A characteristic switch in ER expression and growth between normal and cancer is retained in organoid culture Multicolor imaging of iHBEC^ERpos^ (left column) and MCF7 (right column) plated on a feeder of human fibroblasts and stained after two weeks for MUC1 (green) and keratin K19 (red) on a background of blue nuclei (DAPI) in BBMYAB with TGFβR inhibitors (upper panel) and in the presence of estrogen (middle panel). Whereas iHBEC^ERpos^ cells exhibit outside-in polarization and mostly form acini in the absence of estrogen and more elaborate branching morphogenesis in the presence of estrogen, MCF7 cells under both conditions form irregular colonies without signs of tubular morphogenesis. Dual color imaging of iHBEC^ERpos^ (left column) and MCF (right column) in organoid culture stained for ER (green) and Ki67 (red) (lower panel) show preferential segregation into separate compartments in iHBEC^ERpos^ as opposed to more frequent overlap in MCF7 (orange nuclei).

These differences led us to speculate whether iHBEC^ERpos^ and MCF7 would also recapitulate the widely appreciated dissociation between ER expression and cell proliferation in the normal breast as opposed to its disruption in cancer [32]. For this purpose we plated primary EpCAM^high^/CD271^low^/CD166^high^/CD117^low^ ER-positive cells, iHBEC^ERpos^, and MCF7 on human breast fibroblasts [33]. Whereas iHBEC^ERpos^ under these conditions behaved like EpCAM^high^/CD271^low^/CD166^high^/CD117^low^ primary cells and readily formed correctly polarized K19^+^/K14^-^ acini in the presence of TGFβR inhibitors, and in the presence of estrogen, branching structures, MCF7 formed tumor-like nests of cells without appreciable polarization (Figure 6). In contrast to normal ER^pos^ cells, which do not grow [22] or undergo morphogenesis on fibroblast feeders [33] in the absence of TGFβR inhibitors, growth of MCF7 was inhibited by TGFβR inhibitors. More importantly, we found that in iHBEC^ERpos^ for the major part segregated ER^pos^ cells from Ki-67-positive cells while in MCF7 cells staining frequently overlapped (Ki-67 and ER co-expression in 6.5 % +/- 2.6 versus 32.3 % +/-5.5 of the cells, respectively, Figure 6). Based on these findings, we conclude that together, iHBEC^ERpos^ and MCF7 are well suited for lineage aligned comparisons between normal and cancer within the context of human breast cancer.

## DISCUSSION

By far the majority of cellular turnover in the normal human breast takes place in the luminal epithelial lineage, and with rare exceptions breast cancer – including the so-called basal-like – originates from this lineage [23]. Paradoxically, attempts to model breast homeostasis in cell based assays have been severely confounded by overgrowth of another major lineage, i.e. the myoepithelial [16]. We describe here an established cell line iHBEC^ERpos^ which remain luminal-like in TGFβR2i-1 without the inherent propensity to drift towards the myoepithelial lineage and thus lending itself to more sensible comparisons with breast cancer. A fundamental property that distinguishes this cell line from previous human breast cell lines of non-malignant origin is the expression of functional sex hormone receptors. We here present evidence that the cell line represents a luminal progenitor and that it may serve to unravel the enigmatic division of labor between steroid hormone expressing cells and proliferating cells in the normal breast -perhaps being extrapolatable to all endocrine receptor expressing tissues as opposed to cancer.

Circulating estrogen induces the expression of PR in ER expressing cells and together with progesterone it elicits growth of neighboring luminal progenitors in a paracrine manner ([32];for review see [34]). We find that iHBEC^ERpos^ respond to estrogen by an upregulation of PR and accelerated growth in monolayer culture. With respect to downregulation of ER expression iHBEC^ERpos^ responds very much like the malignant MCF7 cells. However, specifically in MCF7 cells, response to estrogen does not necessarily concur with accelerated growth. In other words, the growth response to estrogen between normal and cancer is not identical. We note that others have reported that MCF7 cells, somewhat dependent on the source of cells, do not respond with growth to added estrogen [35]. *In vivo* ER- positive breast cancer cells are characterized by growth concomitant with ER expression [1, 32]. Our observations indicate that in rBM growth of iHBEC^ERpos^ as opposed to MCF7 cells is regulated in a manner similar to normal breast epithelial cells [24]. However, in spite of recapitulation of acinus-like morphology, the rBM assay does not suffice to maintain ER expression for extended periods. Therefore, iHBEC^ERpos^ serves to dissect both similarities and dissimilarities between normal and cancer.

One of the longstanding puzzles in breast cancer is the apparent dissociation between growth and ER expression in the normal breast and its disruption in cancer [32]. Our findings here of stromal cells as instrumental in providing the necessary microenvironment for maintaining ER expression and segregating ER expressing and growing cells into distinct compartments in iHBEC^ERpos^ opens for a more detailed analysis of the mechanism behind this disruption. One mechanism which is known to function differently in normal versus cancer is TGFβ signaling (for review see [36]). Whereas it in normal breast induces quiescence, in cancer it induces epithelial to mesenchymal transition (EMT). Research by ourselves and others has shown that TGFβ signaling serves to control growth and ER expression of normal mammary epithelial cells [22, 37]. Our findings here that MCF7 exhibits a relatively modest expression of pSMAD2 and fails to respond to TGFβR2i by appreciably modulating ER expression may help explain the disrupted association between growth and ER expression in cancer.

One of the four big questions in the field of breast cancer as recently highlighted in a *Nature* editorial is: “What are the risk factors for the disease?” [38]. It was reasoned that knowledge about susceptibility will illuminate the root causes of this disease and lead to new approaches for prevention and treatment. Accordingly, our findings of a switch in the ER response to TGFβ inhibitors between normal and cancer offers an avenue for a cell based screening of more selective estrogen receptor down regulators (SERDs) in breast cancer chemoprevention.

## MATERIALS AND METHODS

### Ethics statement

Normal breast biopsies of which some were included in previous work [22] were collected with consent from women undergoing reduction mammoplasty for cosmetic reasons. The storage and use of human material has been approved by the Regional Scientific Ethical Committees (Region Hovedstaden, H-2-2011-052) and the Danish Data Protection Agency (2011-41-6722).

### Culture of primary cells and cell lines

EpCAM^high^/CD271^low^/CD166^high^/CD117^low^ ER-positive cells were purified from normal breast as previously described [22]. Cells transduced with hTERT/shp16 in early passage [22] were cultured in Primaria (#3813, Becton Dickenson) in the presence of TGFβR2i medium (Dulbecco's modified Eagle's medium (DMEM, high glucose, no calcium, Life Technologies):Ham's F12 Nutrient Mixture (F12, Life Technologies), 3:1 v/v), 0.5 μg/ml hydrocortisone, 5 μg/ml insulin, 10 ng/ml cholera toxin (Sigma-Aldrich), 10 ng/ml epidermal growth factor (Peprotech), 1.8 x10^-4^ M adenine (Sigma Aldrich), 10 μM Y-27632 (Axon Medchem) and 5% fetal bovine serum (Sigma Aldrich), with the addition of the selective inhibitor of TGF-β type I receptor activin receptor-like kinase ALK5, ALK4 and ALK7, SB431542 (10 μM, Axon 1661, Axon Medchem) and an inhibitor of autophosphorylation of ALK-5, RepSox (25-50μM, R0158, Sigma Aldrich) [22]). To restrict the luminal phenotype, in 6th and again in 11th passage, CD146^high^ cells were purified by FACS (P1H12 1:500, ab24577, Abcam, as primary antibody and goat anti-mouse IgG1 Alexa Flour 647, 1:500, Life Technologies as secondary), and in 22^nd^ passage CD146^high^/CD117^high^ cells (CD146, 1:20, BD Biosciences followed by IgG1 Alexa Flour 647 and CD117, 104D2-PE (1:20)) were sorted. For some experiments cells resorted as EpCAM^+^/CD117^high^ in passage 35 were employed. For dead cell discrimination cells were incubated with either propidium iodide (1μg/ml, Invitrogen) or Fixable Viability Dye eFluor 780 (1:1000, Affymetrix) prior to FACS (FACSAria I and II, BD Biosciences). iHBEC^ERpos^ cells were adapted to grow in modified TGFβR2i medium, TGFβR2i-1, i.e. substitution of epidermal growth factor for amphiregulin (5 nM, R&D Systems or Peprotech) and omission of hydrocortisone and cholera toxin, in passage 27, 29 or 30. To ensure that the cellular origin, EpCAM^high^/CD271^low^/CD166^high^/CD117^low^ cells, could also grow and express the luminal phenotype in TGFβR2i-1, primary cells expanded in TGFβR2i were passaged with TGFβR2i-1 at a density of 12,000 cells/cm^2^, and ER expression was analyzed up to passage four by immunocytochemistry.

MCF7 cells were obtained and cultured as previously described [39]. The cells were adapted to grow on Primaria in TGFβR2i-1 in passage 309. To demonstrate short-term response to TGFβR2i the parental line was seeded in passage 283 at 5,000 cells/cm^2^, counted at day 7, 14 and 21, and the adapted cells grown in TGFβR2i-1 for 37 passages were switched back to TGFβR2i, counted and passed at day 12 and counted at day 26.

Registration of population doublings was started immediately upon switching iHBEC^ERpos^ and MCF7 to TGFβR2i-1 and population doublings were calculated as *n*= 3.32(log UCY-log*I*) +*X*, where *n*= population doubling, UCY = cell yield, *I*= inoculum and *X*= population doubling rate of inoculum.

Normal intralobular fibroblasts were sorted by FACS as CD105^high^/CD26^low^ and cultured as described [33].

### Reconstituted basement membrane (rBM) cultures

To recapitulate *in situ* morphology 400,000 iHBEC^ERpos^ or 200,000 MCF7 cells were embedded in 300 μl ice cold Matrigel^R^ Matrix (growth factor reduced and phenol red free, 356231, Corning), seeded in a 24-well (Nunc) and solidified at 37°C before addition of 1 ml CDM3 [40] without HEPES and trace element mix, in which epidermal growth factor was replaced by amphiregulin (5 nM) and supplemented with TGFβR inhibitors. Morphology was observed and photographed by phase contrast microscopy [24]. Colony formation in two times technical triplicates was quantified by phase contrast microscopy at 20x magnification using an ocular grid, 300 colonies per gel. Gels were frozen in n-hexane cooled in dry ice and prepared for immunostaining after 14 days.

### Co-culture

EpCAM^high^/CD271^low^/CD166^high^/CD117^low^ primary cells, iHBEC^ERpos^ or MCF7 cells were plated at a density of 5,600 cells/cm^2^ on confluent cultures of normal intralobular CD105^high^/CD26^low^ fibroblasts in modified breastoid base medium without HEPES [41] (Dulbecco's Modified Eagle Medium/Nutrient Mixture F-12, 1:1, Life Technologies), 1 μg/ml hydrocortisone, 9 μg/ml insulin, 5 μg/ml transferrin (Sigma-Aldrich), 5.2 ng/ml Na-Selenite (BD Industries), 100 μM ethanolamine (Sigma-Aldrich), 20 ng/ml basic fibroblast growth factor (PeproTech), 5 nM amphiregulin, with the addition of 10 μM Y-27632, 1.8 x10^-4^ M adenine and serum replacement B27 (20μl/ml, Life Technologies) [22] (BBMYAB, [33]). The day after plating, SB431542 and RepSox were added. To assess the influence of estrogen, β-estradiol (10^-8^M, E2758, Sigma-Aldrich) or vehicle (ethanol) was added. Cultures were observed daily by phase contrast microscopy.

### Immunocytochemistry

Cell cultures and 7 μm sections of gels were prepared for immunocytochemical staining essentially as described and the standard fixation protocol is methanol for 5 min at -20°C [21, 42, 43]. Of note, however, staining for ER requires a special fixation protocol. In brief, cultures were rinsed in phosphate buffered saline (PBS), pH 7.4 prior to fixation for 5 min at RT in 3.7% formaldehyde, two rinses in PBS, fixation in methanol:acetone 1:1 v/v for 5 min at -20°C, two rinses in PBS, permeabilization in 0.1% Triton X-100 in PBS, twice for 7 min, rinse in PBS and kept wet prior to application of blocking buffer. To verify the luminal phenotype cells were stained for K19 (BA16, 1:25, Abcam), K8 (TS1, 1:25, Novocastra) and sialomucin MUC1 (115D8, 1:10, Monosan) and to exclude a basal phenotype for K14 (LL002, 1:25, NeoMarkers) and p63 (7JUL, 1:10, Novocastra) for 90 minutes, washed three times in 10% normal goat serum in PBS prior to 30 min incubation with AF488-conjugated secondary antibodies.

To assess polarization in rBM, sections of gels were stained with primary antibodies against sialomucin (1:10) and K19 (1:50, Abcam or 1:800, Genway) for 60 min followed by 60 min with secondary antibodies. Staining for ER was performed using peroxidase (SP1 ready-to-use, Labvision) or fluorescence (1D5 1:25, Dako M7047)).

Co-cultures were double-stained with combinations of K19 (BA16 1:50, Abcam and 1:800, Genway, anti-IgG1 AF568 1:500), K14 (LL002 1:25, anti-IgG3 AF488 1:500), ER (1D5 1:25, anti-IgG1 AF568, 1:500), MUC1 (115D8 1:10, anti- IgG2b AF488, 1:500) and Ki67 (SP6 rabbit monoclonal, 1:25, RM 9106-S, Thermo Scientific, goat anti-rabbit IgG AF488, 1:500) at day 13-14.

Nuclei were stained with 4, 6-diamidino-2-phenylindole (DAPI, Life Technologies) and sections or cultures were mounted with ProLong Gold antifade reagent prior to confocal microscopy (Zeiss, LSM700). The degree of overlap between ER and Ki-67 was assessed by counting 3×200 cells in technical triplicate of each culture on randomly collected microscopic images.

### Response to estrogen

The response to estrogen and an estrogen receptor antagonist was assessed by plating four sets of 6,000 iHBEC^ERpos^ cells/cm^2^ or 4,000 MCF7 cells/cm^2^ and exposing them to estrogen (10^-8^M, β-estradiol, E2758, Sigma-Aldrich) with or without estrogen receptor antagonist (10^-8^M and 10^-9^M Fulvestrant, ICI 182,780, Sigma-Aldrich, respectively) as compared to vehicle (ethanol) for seven days prior to trypsination and counting (CASY cell counter). An additional set of cultures was stained for ER and PR by peroxidase and counterstained with hematoxylin. To test the response to estrogen with or without ICI 182,780 (10^-7^M) in the parental MCF7 line in standard medium, lines in passage 287 and 312, cultured in separate laboratories since passage 240 were employed. To quantify for ER and PR expression upon estrogen stimulation 12,000 iHBEC^ERpos^ cells/cm^2^ in passage 35 (adapted to TGFβR2i-1 in passage 29) or 5,000 MCF7 cells/cm^2^ in passage 328 (adapted in passage 309) were cultured with vehicle or estrogen (10^-8^M) for 8 days and stained by peroxidase, counterstained with hematoxylin and quantified (3×100 cells) [22].

### RNA extraction, real-time quantitative (RT-q)PCR and transcriptome analysis

Prior to comparison of transcriptional profiles in response to estrogen, iHBEC^ERpos^ cells and MCF7 cells were cultured in TGFβR2i -1 for a total of 32 and 45 days, respectively. iHBEC^ERpos^ cells in passage 33 were plated at a density of 24,000 cells/cm^2^ and MCF7 in passage 316 at a density of 4,000 cells/cm^2^. After six days of culture, cells were exposed to estrogen (10^-8^M) or vehicle (ethanol) for 1, 3, 6, 12 and 24 hours. Total RNA was extracted and reverse transcribed and RT- qPCR was performed as described [22].

For transcriptome analysis each group with or without estrogen for 6h was run in triplicate using RNA-Seq technology [44]. Sequencing and bioinformatics analysis was conducted by Beijing Genomics Institute (BGI), Hongkong. In short, Oligo dT magnetic beads were used to select mRNAs with poly A tails or DNA probes were used to hybridize rRNAs to get rid of rRNAs. Selected mRNAs were fragmented and reversely transcribed to double-stranded cDNA (dscDNA) by N6 random primers. Ends of dscDNAs in turn were repaired with phosphate at 5′ end and A at 3′ end in order to ligate adaptors with stickiness T at 3′ end to the dscDNAs which were subjected to amplification. In order to prepare products for sequencing, called a library, the PCR products were denatured and single stranded PCR products were cyclized by splint oIigos with DNA ligase. The prepared library was then sequenced using BGISEQ-500 platform generating 23,958,189 raw sequencing reads. Clean reads of 23,950,466 after filtering low quality was mapped to reference using HISAT/Bowtie2 tool with the mean mapping rate of 79%. Gene quantification was measured by FPKM calculated based on the expectation maximization algorithm called RSEM ([45].

For subcellular classification, transcribed genes found here without estrogen stimulation in iHBEC^ERpos^ and MCF7, respectively, were compared to a previously published dataset of human breast lineage gene expression profiles [23]. Differentially expressed gene lists of the human breast lineages defined by CD49f and EpCAM status based on microarray analysis was obtained from supplementary tables 5, 6 and 7 in Lim et al. [23]. Among them, the most highly expressed 20 genes in each lineage: MaSC, luminal progenitor (pLs), or mature luminal (mLs) were selected and searched for their presence in our RNA-Seq data. Using gene expression levels calculated by FPKM from the clean data with coverage>0 in triplicate, 12 genes in MaSc, 14 genes in pLs and 12 genes in mLs were present in our sequencing data.

To identify differentially expressed genes regulated by estrogen, the NOIseq method [25] was performed, using the filtering condition of the probability higher than 0.7 with fold difference more than 2. When FPKM value was not available (coverage=0), the value was treated as 0.01 as the default FPKM. Among statistically significant differentially expressed genes, we selected a repertoire of genes that were upregulated either in iHBEC^ERpos^ or in MCF7. Differentially regulated genes in MCF7 were further validated by comparing to a previously published dataset on estrogen-regulated genes in MCF7 [28].

### Western blotting

For Western blotting protein was extracted at day 6 from iHBEC^ERpos^ cells in passage 36 seeded at 18,000 cells/cm^2^ and MCF7 cells in passage 325 seeded at 4,000 cells/cm^2^ and cultured with or without TGFβR inhibitors. 25 μg of protein was loaded in each lane and Western blotting was performed as previously described [22].
